# Valsalva-Related Subretinal Hemorrhage as a Presenting Symptom of Polypoidal Choroidal Vasculopathy

**DOI:** 10.1155/2017/9650287

**Published:** 2017-09-12

**Authors:** Yousif Subhi, Torben L. Sørensen

**Affiliations:** ^1^Clinical Eye Research Division, Department of Ophthalmology, Zealand University Hospital, Roskilde, Denmark; ^2^Faculty of Health and Medical Sciences, University of Copenhagen, Copenhagen, Denmark

## Abstract

**Purpose:**

To describe a case of Valsalva-related subretinal hemorrhage as a presenting symptom of polypoidal choroidal vasculopathy (PCV). The patient refrained from treatment against our best advice, and thus this is also a rare case of the natural course of an untreated PCV.

**Methods:**

Case report.

**Results:**

A 66-year-old female with a respiratory infection coughed intensely until exhaustion, after which she developed visual symptoms on the right eye. Primary care ophthalmologist examined the patient on the same day of the onset of symptoms and referred her to our tertiary medical retinal service for detailed retinal diagnosis including fluorescein and indocyanine green angiography. The right eye had a large subretinal hemorrhage and pigment epithelium detachment in the lower temporal arcade with foveal involvement. Against our best advice, the patient refused treatment. In the following 9 months, the BCVA decreased from 68 to 55 ETDRS letters, the subretinal hemorrhage almost regressed, pigment epithelium detachments persisted, and macular edema, intraretinal cysts, and subretinal fibrosis developed.

**Conclusions:**

Although classic Valsalva retinopathy with preretinal hemorrhage in most cases can be managed by careful observation and no treatment, this case demonstrates that Valsalva-related subretinal hemorrhage needs different attention and approach.

## 1. Introduction

Coughing induces a Valsalva-like situation: a deep breath in, the glottis shuts, the diaphragm presses against the lungs, and the glottis opens blasting out air. The change in intrathoracic pressure is 100–250 mmHg depending on the violence of the cough [1]. This reduces cardiac preload, increasing the venous pressure. First described by Duane in 1972 [2], the Valsalva retinopathy classically describes rupture of small retinal capillaries leading to a preretinal hemorrhage. Noncomplicated cases are managed by careful observation, as the hemorrhage resolves within months where visual acuity gradually improves, in most cases into complete recovery [3–5]. Here, we describe a case of Valsalva-related hemorrhage of subretinal origin, which turned out to be the presenting symptom of polypoidal choroidal vasculopathy (PCV), a disease characterized by polypoidal dilations in the choroid.

## 2. Case Presentation

A 66-year-old female with a respiratory infection decided to “get rid of it the phlegm once for all” by coughing strongly and intensely until exhaustion. During the following hour, the patient acutely developed blurred vision and light flashes on the right eye. Primary care ophthalmologist examined the patient on the same day of the onset of symptoms and referred her to our tertiary medical retinal service. Our examination included slit-lamp biomicroscopy, fluorescein and indocyanine green (ICG) angiography, spectral-domain optical coherence tomography, and B-scan ultrasound. Best-corrected visual acuity (BCVA) of each eye was measured using the Early Treatment of Diabetic Retinopathy Study (ETDRS) chart. The patient did not have any comorbidities. Only relevant family history was a now deceased father, who had received intravitreal injection treatments for a disease unknown to the patient.

**Figure 1:**
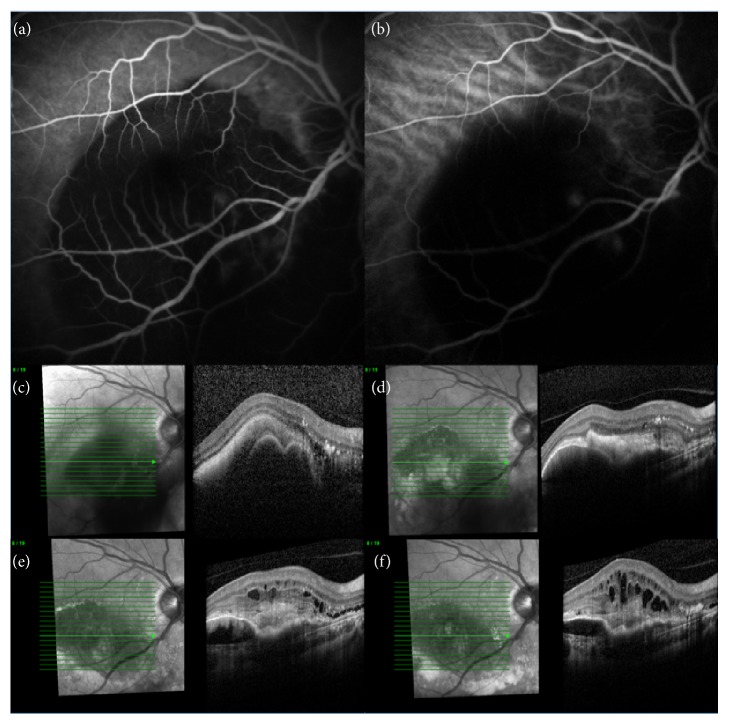
Retinal angiography using fluorescein (a) and indocyanine green (b) of the patient's right eye. The polypoidal lesions are difficult to visualize due to the massive subretinal hemorrhage. Optical coherence tomography at (c) baseline (BCVA: 68 ETDRS letters, Snellen 6/15), (d) 3 months (BCVA: 64 ETDRS letters, Snellen 6/19), (e) 6 months (BCVA: 55 ETDRS letters, Snellen 6/24), and (f) 9 months follow-up (BCVA: 55 ETDRS letters, Snellen 6/24) shows a gradual development of subretinal fibrosis and intraretinal cysts.

The right eye had subretinal hemorrhage and pigment epithelium detachment in the lower temporal arcade with foveal involvement, and the retinal angiography showed two hyperfluorescent spots on early-phase ICG but the images were somewhat blurred due to the large subretinal hemorrhage (Figure 1). Intraocular pressure was normal (16 mmHg) and ultrasound found no evidence of tumor. On OCT images, we found polyp-like retinal pigment epithelium elevations (Figure 1). The BCVA was 68 ETDRS letters (Snellen = 6/15). The left eye was without any pathologies or drusen and the BCVA was 85 ETDRS letters (Snellen = 6/6).

Against our best advice, the patient initially refused commencement of intravitreal anti-VEGF treatment. Photodynamic therapy was not possible due to risk of hemorrhage. We invited the patient for follow-ups with 3-month intervals. During the total follow-up of 9 months, the BCVA decreased to 55 ETDRS letters (Snellen = 6/24); the subretinal hemorrhage almost regressed; pigment epithelium detachments persisted; and macular edema, intraretinal cysts, and subretinal fibrosis developed (Figure 1).

## 3. Discussion

Valsalva-related preretinal hemorrhages are commonly caused by macroaneurisms, and subretinal hemorrhages are commonly seen in choroidal neovascularizations and especially in cases with retinal angiomatous proliferation. We here describe a case report of PCV that present with Valsalva-induced acutely developed subretinal hemorrhage. Although classic Valsalva retinopathy with preretinal hemorrhage in most cases can be managed by careful observation, this case demonstrates that subretinal hemorrhages need different attention and approach.

PCV is similar to neovascular age-related macular degeneration (AMD) in being a disease of the posterior section with a significant choroidal component. It differs from neovascular AMD in being characterized by polypoidal vascular dilations with or without an associated branching choroidal network, by not being associated with drusen maculopathy, and by often presenting with hemorrhage [6, 7]. This is also what we see in our case of PCV with polypoidal dilations and not a single drusen in the fellow eye. The polypoidal dilations can manifest clinically as quiescent polyps in the absence of subretinal or intraretinal fluid or hemorrhage; as exudative polyps with exudations leading to intraretinal cysts, subretinal fluids, and pigment epithelium detachments; or as hemorrhagic polyps with subretinal hemorrhage or subretinal pigment epithelium hemorrhage that also may include any exudative characteristics. The latter, hemorrhagic type, seems to be the most prevalent [6]. Using our case as an example, we propose that, in some cases, quiescent polypoidal lesions may persist asymptomatic until vascular-originated or mechanical reasons for developing large lesions of a more symptomatic characteristic. Our patient probably had quiescent polypoidal lesions without any symptoms until the Valsalva-induced development of the subretinal hemorrhage. However, it must be stressed that hemorrhagic presentation of PCVs can occur even in the absence of Valsalva, and a cause of effect relationship in this case report is only suspected due to a close temporal association. Studies exploring the details of initial presentation and progression of PCV are needed.

Since the diagnosis of PCV is dependent on retinal angiography, population-based studies on PCV epidemiology are rare. In Asians, PCV accounts for approximately half of all patients with neovascular AMD [6], while in Caucasians the prevalence estimates are around 8% [6]. As such, it is a relatively rare disease in a Danish population. Smoking is a strong risk factor (OR 4.4) [6], but otherwise little is known. Abnormal extracellular matrix homeostasis may play a role as suggested by experimental animal studies and human serum analyses [6], but the overall picture remains unclear. From this case point of view, if the polyps are generally surrounded by a weak and fragile extracellular environment, it seems reasonable that Valsalva-like events cause subretinal hemorrhages in cases with PCV.

The natural history of untreated PCV is vision loss and scarring with hemorrhage as a main reason [8]. Hemorrhagic PCVs must be treated from the outset, even in suspicious PCV cases, since otherwise the final visual outcome will be poor [8]. Although our patient at the end of our follow-up decided to start anti-VEGF treatment, the game may be over when fibrosis and scarring develop, and it is important to remember that timely initiation of treatment and compliance to treatment are important factors that influence outcomes from anti-VEGF treatment [9, 10]. Subretinal hemorrhage damages retinal tissue and should be avoided. Although anti-VEGF treatment of PCV does provide good clinical results [11], management of subretinal hemorrhage can also be achieved at an acceptable level using pneumatic displacement and tissue plasminogen activator to remove remaining blood clots [12].

## References

[1] Sharpey‐Schafer E. P. (1953). Effects of coughing on intra‐thoracic pressure, arterial pressure and peripheral blood flow. *The Journal of Physiology*.

[2] Duane T. D. (1972). Valsalva hemorrhagic retinopathy. *Transactions of the American Ophthalmological Society*.

[3] Shukla D., Naresh K. B., Kim R. (2005). Optical coherence tomography findings in Valsalva retinopathy. *American Journal of Ophthalmology*.

[4] Tildsley J., Srinivasan S. (2009). Valsalva retinopathy. *Postgraduate Medical Journal*.

[5] Choudhry N., Rao R. C. (2014). Images in clinical medicine. valsalva retinopathy. *The New England Journal of Medicine*.

[6] Wong C., Wong T., Cheung C. (2015). Polypoidal choroidal vasculopathy in Asians. *Journal of Clinical Medicine*.

[7] Chan W.-M., Lam D. S. C., Lai T. Y. Y. (2004). Photodynamic therapy with verteporfin for symptomatic polypoidal choroidal vasculopathy: one-year results of a prospective case series. *Ophthalmology*.

[8] Cheung C. M. G., Yang E., Lee W. K. (2015). The natural history of polypoidal choroidal vasculopathy: a multi-center series of untreated Asian patients. *Graefe's Archive for Clinical and Experimental Ophthalmology*.

[9] Rasmussen A., Brandi S., Fuchs J. (2015). Visual outcomes in relation to time to treatment in neovascular age-related macular degeneration. *Acta Ophthalmologica*.

[10] Subhi Y., Sørensen T. L. (2017). Neovascular age-related macular degeneration in the very old (≥90 years): epidemiology, adherence to treatment, and comparison of efficacy. *Journal of Ophthalmology*.

[11] Gharehbagh S. S., Subhi Y., Sørensen T. L. (2017). Efficacy of aflibercept for polypoidal choroidal vasculopathy in Caucasians. *Acta Ophthalmologica*.

[12] Olivier S., Chow D. R., Packo K. H., MacCumber M. W., Awh C. C. (2004). Subretinal recombinant tissue plasminogen activator injection and pneumatic displacement of thick submacular hemorrhage in Age-Related macular degeneration. *Ophthalmology*.

